# Molecular chaperones: Guardians of tumor suppressor stability and function

**DOI:** 10.18632/oncotarget.28653

**Published:** 2024-10-01

**Authors:** Jennifer A. Heritz, Sarah J. Backe,, Mehdi Mollapour

**Affiliations:** ^1^Department of Urology, SUNY Upstate Medical University, Syracuse, NY 13210, USA; ^2^Upstate Cancer Center, SUNY Upstate Medical University, Syracuse, NY 13210, USA; ^3^Department of Biochemistry and Molecular Biology, SUNY Upstate Medical University, Syracuse, NY 13210, USA; ^4^Syracuse VA Medical Center, New York VA Health Care, Syracuse, NY 13210, USA

**Keywords:** molecular chaperone, tumor suppressor, renal cell carcinoma, Birt-Hogg-Dubé (BHD) syndrome, TSC syndrome

## Abstract

The term ‘tumor suppressor’ describes a widely diverse set of genes that are generally involved in the suppression of metastasis, but lead to tumorigenesis upon loss-of-function mutations. Despite the protein products of tumor suppressors exhibiting drastically different structures and functions, many share a common regulatory mechanism—they are molecular chaperone ‘clients’. Clients of molecular chaperones depend on an intracellular network of chaperones and co-chaperones to maintain stability. Mutations of tumor suppressors that disrupt proper chaperoning prevent the cell from maintaining sufficient protein levels for physiological function. This review discusses the role of the molecular chaperones Hsp70 and Hsp90 in maintaining the stability and functional integrity of tumor suppressors. The contribution of cochaperones prefoldin, HOP, Aha1, p23, FNIP1/2 and Tsc1 as well as the chaperonin TRiC to tumor suppressor stability is also discussed. Genes implicated in renal cell carcinoma development—*VHL*, *TSC1/2*, and *FLCN*—will be used as examples to explore this concept, as well as how pathogenic mutations of tumor suppressors cause disease by disrupting protein chaperoning, maturation, and function.

## INTRODUCTION

Genes related to oncogenesis can generally be divided into two categories: proto-oncogenes and tumor suppressor genes. Proto-oncogenes are typically involved in signaling pathways that promote cellular growth and only promote tumorigenesis upon aberrant gain-of-function mutations, consequently referred to as oncogenes [1]. In opposition, tumor suppressor genes are generally involved in the suppression of cell growth and upon loss-of-function alterations lead to tumorigenesis [1]. Mutations in tumor suppressor genes are recessive and follow the pattern of Knudson’s two-hit hypothesis [2, 3]. This refers to both of the alleles of a tumor suppressor gene needing to be inactivated in order for the cell to become cancerous [3]. The first mutation, or “hit”, can be sporadic (acquired) or inherited (germline) to cause heterozygosity of the tumor suppressor gene [2]. This heterozygosity predisposes individuals for tumor development, as the spontaneous loss or inactivation of the second allele (“second-hit”) or “loss of heterozygosity” may trigger oncogenesis [2].

Pathogenic mutations in tumor suppressor genes exist within protein-coding exons and cause disease by inhibiting the normal function of the encoded proteins. However, non-pathogenic mutations, known as polymorphic variations, may occur in the non-coding regions of DNA [4]. Though polymorphic variations can coexist with the disease phenotype, they are not the cause [4]. Consequently, it is difficult to determine if a mutation found in the sequence from a tumor is a pathogenic or a polymorphic variation without further experimental data [4]. Therefore, active research is ongoing to dissect the molecular mechanisms of proposed pathogenic mutations toward promoting oncogenesis.

Polypeptides form intramolecular contacts according to their amino acid sequence that drive the folding of a protein into its native and functional state with the least amount of free energy [5, 6]. However, the crowded nature of the cellular environment and the larger, multi-domain nature of many proteins makes it impossible for them to fold on their own on a biologically relevant timescale [7, 8]. Partially folded intermediates or misfolded proteins are not only non-functional, but also have a tendency to form toxic aggregates that can induce proteotoxic cellular death [6]. Thus, proteins outside of their native state are subject to removal through the ubiquitin-proteosome or autophagy-lysosomal systems. The cell ensures the stability and efficient folding of proteins into native states through the molecular chaperone system. This is comprised of a complex network of chaperones, cochaperones, and chaperonins working in concert to fold newly-synthesized polypeptides from the ribosome, shield partially-folded intermediates from removal, refold aberrant proteins, and ensure that proteins damaged beyond repair are degraded [6, 7, 9, 10]. Sequential interactions of chaperone ‘clients’ with multiple members of this network provides full control of functional protein levels based on the needs of the cell, allowing tight regulation of physiological pathways.

Though broadly grouped together, tumor suppressors are highly diverse, with a wide variety of functions, structures, and cellular localizations [11]. Despite this diversity, the number of tumor suppressors found to be regulated by the chaperone network continues to grow [12]. This dependance on molecular chaperones therefore represents a rare commonality of tumor suppressors.

## THE MOLECULAR CHAPERONE NETWORK

**Figure 1:**
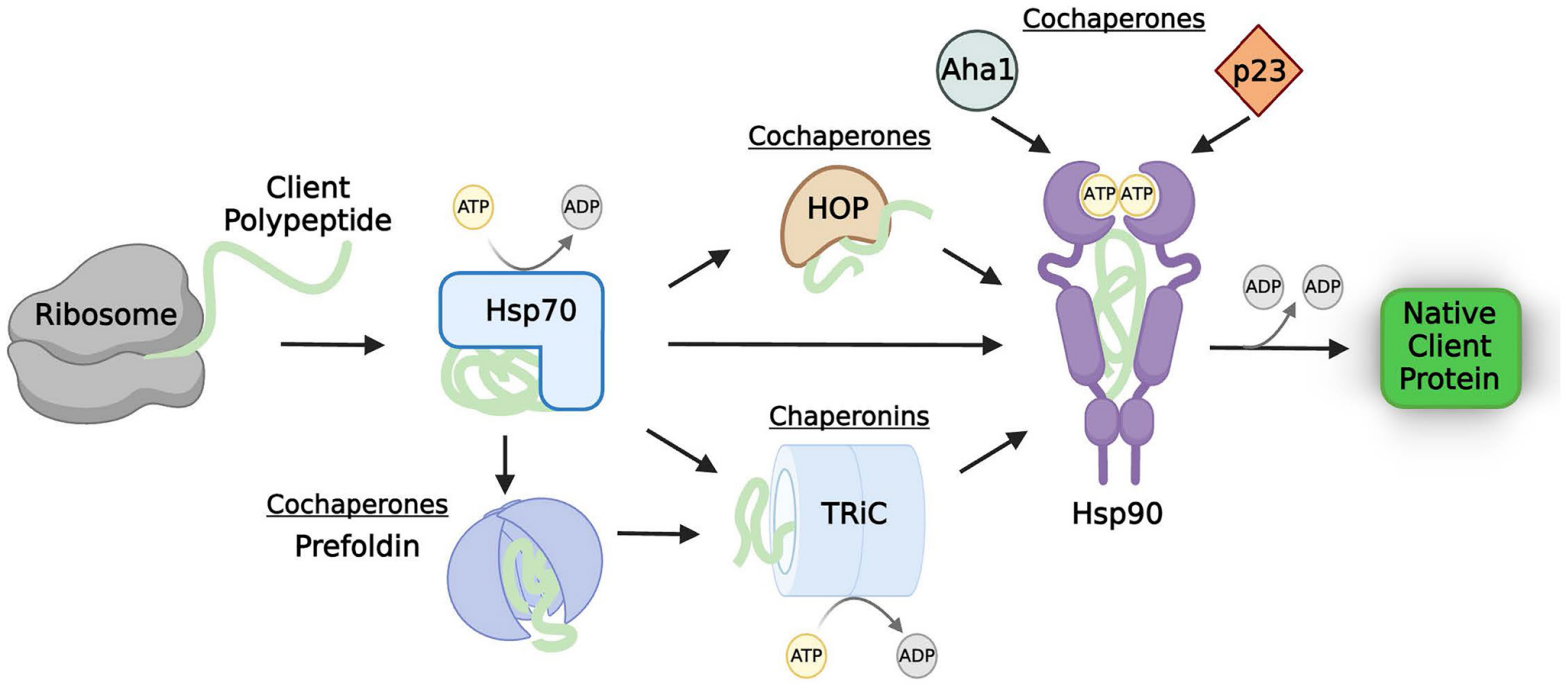
The molecular chaperone network. Client proteins rely on sequential interactions with chaperones, cochaperones, and chaperonins to fold into an active, native, state. Thus, functional levels of client proteins are tightly regulated by this process. The chaperones, cochaperones, and chaperonins commonly involved in the chaperoning of tumor suppressor proteins are shown; however, the exact number and types of molecular chaperone network members necessary for proper chaperoning is client-specific. Abbreviations: Hsp70: heat-shock protein 70; HOP: Hsp70-Hsp90 organizing protein; TRiC: tailless complex polypeptide 1 ring complex; Hsp90: heat-shock protein 90; Aha1: Activator of Hsp90 ATPase.

Several distinct classes of molecular chaperones exist to achieve protein homeostasis, or proteostasis, for their diverse set of clients. The heat-shock proteins (Hsps) describe a class of highly conserved chaperones typically classified according to their molecular weight, including but not limited to, Hsp70, Hsp90, and Hsp60 (chaperonins) that mainly participate in protein folding/refolding [6, 13]. Hsps weakly bind to clients that can “sample” multiple states—unfolded, quasi-native, and native [14–16]. Generally, this transient binding of the partially unstructured client to the chaperone protects the client while promoting this “sampling”, driving it to fold [17–19]. However, the exact dynamics of these interactions are client and chaperone specific, and even two proteins with high sequence and structure similarity can be clients of different chaperones [20]. Cochaperones are binding partners of chaperones whose stability do not depend on chaperones themselves. Cochaperone activity, ATP binding, and post-translational modifications (PTMs) together form a complex hierarchy that regulates chaperone client binding and release cycles [21–23]. Generally, Hsp70 interacts with newly-synthesized polypeptides from the ribosome first (Figure 1) [6, 18, 24–26]. The ATP-bound open state of Hsp70 allows the binding of the client before hydrolysis triggers the closing of the conformation around the client [10, 27–30]. In this bound state, the polypeptide is protected from cellular removal and has a few seconds to correctly fold. Nucleotide exchange factor binding to Hsp70 catalyzes ADP/ATP exchange to open Hsp70, releasing the folded protein [28, 30, 31]. Proteins that are not fully matured by interaction with Hsp70 may be transferred to chaperonins or to Hsp90 [6, 18, 32].

The tailless complex polypeptide 1 ring complex (TRiC) chaperonin is a large, multimeric complex that fully encapsulates client proteins, which are typically larger, multi-domain proteins [33, 34]. Prefoldin, a cochaperone of TRiC, mediates the ‘loading’ of some clients to TRiC (Figure 1) [7, 35]. Like Hsp70, TRiC binding to clients is ATP regulated, and clients enter its ‘cage’ one molecule at a time to avoid misfolding [6]. The specialized inner wall is hydrophilic and negatively charged [32, 36]. Hydrolysis of the bound ATP molecules sets the biological timer for how long the client protein is enveloped. Once fully hydrolyzed to ADP, the TRiC complex opens to release the client. Envelopment of these proteins into the specialized interior of the chaperonin cage can either passively prevent aggregation or accelerate correct folding [37–39].

Hsp90 functions downstream of Hsp70 (and TRiC, if necessary) and its activity is also regulated by cochaperones [17, 18, 40]. Though there are currently about fifty identified Hsp90 cochaperones, three are the most common across different client types: Hsp70-Hsp90 organizing protein (HOP), activator of Hsp90 ATPase (Aha1), and p23 [40, 41]. HOP mediates the transfer of some clients from Hsp70 to the open conformation of Hsp90 (Figure 1) [42, 43]. Aha1 binds to Hsp90 along with ATP, displacing HOP and closing the Hsp90 dimers around the client protein [44, 45]. The cochaperone p23 displaces Aha1 to stabilize the “closed and twisted” conformation of Hsp90, allowing the maturation of the enclosed client [41, 46]. Upon completion of ATP hydrolysis, Hsp90 opens to release the active client. In this way, cochaperones provide directionality to the Hsp90 cycle [41, 47]. The ATPase activity of Hsp90 can either be accelerated (i.e., Aha1 binding) or decelerated (i.e., p23 binding) by cochaperones (Figure 1) [48–50]. The folding time required in the cycle is largely client-specific; therefore, cochaperone activity is crucial to control individual client stability and activity [51].

## TUMOR SUPPRESSORS INVOLVED IN RENAL CELL CARCINOMA

Renal cell carcinoma (RCC) is a heterogenous group of diseases stemming from mutations in at least 17 different genes, the majority of which are tumor suppressors [52]. Pathogenic mutations of these genes are typically associated with histologically distinct tumors that respond differently to therapies. Notably, several of the associated protein products rely on the chaperone system [53–56]. This review will discuss three examples of chaperone-dependent tumor suppressors involved in RCC: von Hippel-Lindau (VHL), Tuberous Sclerosis Complex 1 and 2 (TSC1/2), and folliculin (FLCN). Loss of the *VHL* tumor suppressor gene causes the most common and aggressive subtype of renal cell carcinoma, clear cell renal cell carcinoma (ccRCC) [57–60]. Inactivating mutations in either of the TSC genes, *TSC1* and *TSC2*, result in renal angiolipoma (AML). Mutations in the tumor suppressor *FLCN* are associated with multiple histological subtypes of RCC, such as chromophobe, clear cell, oncocytoma, and hybrid oncocytic [61]. Emerging evidence has uncovered that multiple pathogenic mutations in these tumor suppressor genes cause disease by disrupting critical chaperoning pathways, which prevents full maturation of the associated protein and results in loss of function.

### Chaperoning of the tumor suppressor Von Hippel-Lindau

**Figure 2:**
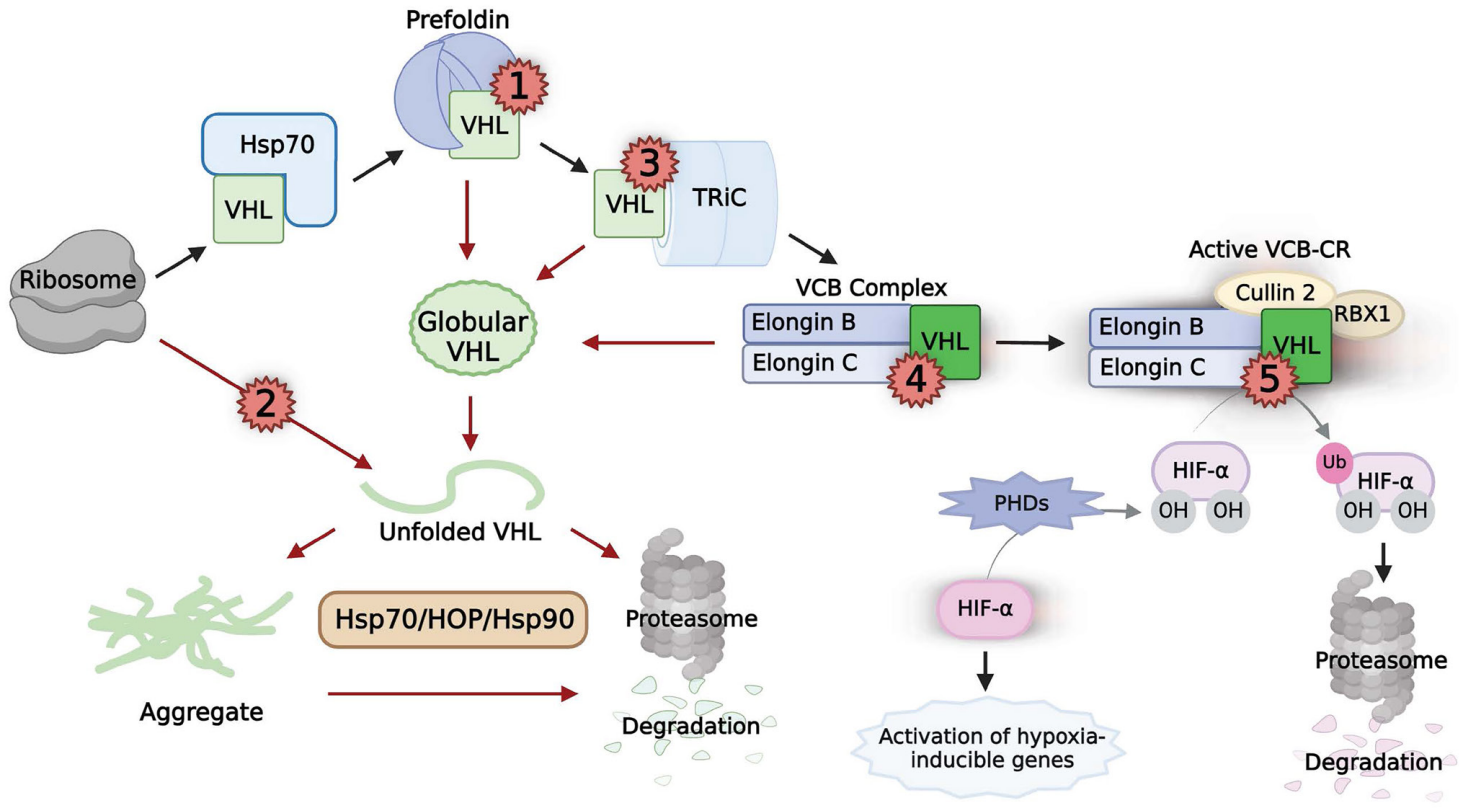
The chaperoning, activity, and pathogenic mutations of VHL. Black arrows indicate the normal chaperoning of VHL. Naϊve VHL (light green box) is chaperoned by the sequential interactions of Hsp70, prefoldin, and TRiC before folding into its native state (neon green box) upon binding to elongins B and C. Subsequent binding of cullin 2 and RBX1 result in a functional VCB-CR complex that regulates HIF-α function. Red arrows indicate pathways caused by multiple classes of pathogenic mutations on VHL (red numbers). Class 1 mutations interrupt prefoldin binding. Class 2 mutations disrupt necessary structures in VHL, directly destabilizing the protein. Class 3 mutations prevent TRiC binding. Class 4 mutations prevent binding to either elongin B or C. Classes 1–4 result in un-chaperoned globular VHL, which can unfold and aggregate. The proteasomal degradation of unfolded VHL and aggregated VHL is reliant on Hsp70, HOP, and Hsp90. Class 5 mutations abrogate the ability of VHL in the VCB-CR complex to bind to HIF-α, rendering the complex non-functional.

VHL, the protein product of the *VHL* gene, has multiple interactors and proposed functions in healthy cells including regulating the cell cycle and maintaining the extracellular matrix through the regulation of fibronectin [53, 62]. However, the most characterized function of VHL in ccRCC is the regulation of the hypoxia-inducible factor (HIF) transcription complexes [63]. The HIF transcription complex binds to DNA to activate numerous genes that promote angiogenesis and anaerobic metabolism [64]. In cells with active VHL, these genes—such as vascular endothelial growth factor (*VEGF*) and glucose transporter-1 (*GLUT1*)—are only activated in hypoxic conditions as an adaptation to reduced oxygen availability [64]. Mechanistically, the HIF complexes HIF-1, HIF-2, and HIF-3 are heterodimers with an α subunit (HIF-1α, HIF-2α, or HIF-3α) and a common HIF-1β subunit [65]. VHL associates with elongins B and C to form the VCB complex, which interacts with culllin 2 and Rbx1 to form the VCB-CR complex that has E3 ubiquitin ligase activity (Figure 2) [53]. VHL acts as the substrate recognition component of VCB-CR that specifically targets each of the three HIF-α subunits (or HIF-α) for ubiquitination and subsequent proteasomal degradation [53, 66–69]. However, for recognition by the VHL ubiquitin ligase complex, the HIF-α subunit must be hydroxylated on one or both of two conserved proline residues. Under normoxia, HIF-α is hydroxylated by prolyl hydroxylase 1 (PHD1), PHD2 and PHD3. Thus, HIF-α expression remains low under normoxic conditions by functional VHL [53]. The hydroxylation by PHD1, PHD2 and PHD3 requires oxygen, so HIF-α cannot be recognized by the VCB-CR under hypoxia. With HIF-α escaping ubiquitination, the transcription factors accumulate and form heterodimers with HIF-1β [53]. These functional heterodimers translocate to the nucleus where they bind to hypoxia-response elements (HRE) to induce transcription of target genes [53].

Individuals with a single mutant or inactivated *VHL* allele have VHL disease, a condition associated with benign vascular tumors of the central nervous system (haemangioblastomas), retina, and adrenal gland (phaeochomocytomas), as well as renal and pancreatic cysts [53, 70, 71]. The development of ccRCC occurs when the second, wild-type allele is spontaneously inactivated or lost [3, 72–74]. VHL-mutant tumors are highly vascularized by the overproduction of the hypoxia-inducible factors, such as VEGF [75–77]. This vascularization promotes tumor growth by delivery of oxygen and nutrients, the removal of waste, and the promotion of metastasis [78].

Although all three members of the HIF-α family are activated by hypoxia and regulated by VHL in the same manner, they are not functionally redundant [79–81]. The many alternative splicing products of HIF-3 are less characterized than the other isoforms, with the products appearing to have conflicting downstream functions [81, 82]. Between HIF-1 and HIF-2, the main driver of RCC appears to be organism and stage-dependent [53, 80, 83–85]. Evidence shows that HIF-1 promotes renal carcinogenesis in mice and early lesions in humans, but HIF-2 is highly enriched and predominantly promotes growth in late-stage human cysts and tumors [83, 86–88].

Thus, the maintenance of functioning VHL is of major interest to the cell, and a major mode of regulation is the chaperoning process. Stability of newly-synthesized VHL relies on sequential cooperation of the chaperone Hsp70 and the chaperonin TRiC (Figure 2). Hsp70 first stabilizes newly-translated VHL before transferring it to TRiC [89]. The involvement of the cochaperone prefoldin as an intermediate step to assist VHL’s transfer to TRiC was somewhat debated [89–91]; however, more recent evidence in human cells demonstrated that interaction with prefoldin does in fact contribute to VHL stability [92, 93]. TRiC is required to stabilize monomeric VHL, which exists in a partially-unfolded molten globule state until interaction with the elongins [93, 94]. Existing briefly in this structurally versatile state may allow VHL to carry out its multiple cellular functions; however, without any binding partners, the naïve protein is unstable and aggregate prone [93–95]. VHL is stabilized into its native state by TRiC-mediated delivery to elongins B and C, and the elongins B and C are reciprocally stabilized through their interactions with each other and VHL [90, 92]. This VCB complex then forms the active VCB-CR E3 ubiquitin ligase upon interaction with culllin 2 and Rbx1 [89, 90, 96]. Thus, the correct sequential chaperoning of VHL is necessary to avoid further unfolding or aggregation, which results in rapid degradation [89, 93, 94].

The degradation of VHL is also regulated by chaperones (Figure 2). Importantly, this is a distinct pathway from its folding. Such a distinction adds an additional layer of cellular control over the physiological triage decision. Hsp70 is required for VHL degradation, but TRiC is not [97]. Furthermore, Hsp90 is required for the degradation pathway, but not the folding pathway [97]. Collectively, the chaperone machinery and binding partners of VHL work in concert to maintain a dynamic equilibrium of the folding and degradation pathways. Correctly semi-folded VHL bound to TRiC will preferentially associate with the elongins to form the VCB complex, and subsequently the functional VCB-CR complex [97]. However, Hsp90 may recognize aberrant conformations of VHL that cannot properly bind to elongins B and C. The cochaperone HOP mediates the transfer of these failed folding intermediates from Hsp70 to Hsp90, which shuttles them to the degradation pathway [97]. The balance between VHL activation and degradation is critical to maintain healthy HIF-1α regulation.

### Pathogenic mutations in *VHL*

The wide phenotypic range of VHL disease has given rise to patient classification based on genotype-phenotype associations [53, 70]. Patients are stratified by the absence (Type 1) or presence (Type 2) of pheochromocytomas. Type 2 is further categorized based into type 2A (with pheochromocytomas and haemangioblastoma), type 2B (with pheochromocytomas, haemengioblastoma, and ccRCC) and type 2C (with pheochromocytomas only). Importantly, the risk of developing ccRCC correlates with the ability of mutant VHL to regulate HIF activity [53]. Types 1 and 2B, the subtypes at high risk of ccRCC development, exhibit gross overexpression of HIF-1α, while the other subtypes without RCC have a much milder overexpression or regular levels [53].

Despite our currrent understanding of the VHL pathway, a major outstanding question remains: how do pathogenic mutations of *VHL* cause tumorigenic loss of HIF regulation? Addressing this question is crucial for the treatment of ccRCC. Feldman and colleagues defined three classes of pathogenic mutations in VHL based on their mechanism of VHL inactivation [98]. Twenty years later, the data show more diversity, and can be divided into two additional classes for a total of five pathogenic mutation classes (Figure 2). Classes 1–4 promote rapid degradation through a chaperone-mediated pathway, depleting the protein levels of VHL to a non-functional level. Futhermore, mutation Classes 2 and 3 directly interfere with chaperone-mediated protein folding. This underscores the fundamental role chaperones play in the regulation of tumor suppressors.

Of the five pathogenic muation classes, the only one that allows correct folding of VHL is Class 5. Class 5 mutations occur in residues that are required for VHL to bind to HIF-α, rendering correctly-formed VCB-CR complexes non-functional [98, 99]. These mutations, such as Y98N and Y112N, are frequently found in VHL disease Type 2B (high risk of ccRCC) [90, 99, 100].

In contrast to Class 5 mutations, many pathogenic mutations in VHL lie outside of the HIF-1α interacting site, causing disease by destabilizing VHL in a variety of mechanisms [99]. Class 1–4 mutations all promote the misfolding or unfolding of monomeric VHL, leading to aggregation before the Hsp90-dependant degradation pathway, or degradation directly (Figure 2) [94]. These pathogenic mutations result in insufficient functional VHL to maintain healthy regulation of HIF-α. Class 1 VHL mutations disrupt the binding of the newly-synthesized VHL protein with prefoldin. A tumorigenic mutation hotspot in VHL has been predicted to be the region responsible for prefoldin binding [101]. This region, at the junction of exon2 and exon3, was confirmed to be responsible for the binding of all six subunits of the prefoldin complex in mammalian cells using a proximity-dependent biotin identification (BioID) screen [92]. Notably, downregulation of the prefoldin complex through the silencing of subunit PFDN3 resulted in reduced wild-type VHL levels in HeLa cells [92]. This is in line with the finding that VHL, unable to associate with prefoldin, was subject to proteasomal degradation [93]. Furthermore, analysis of The Cancer Genome Atlas (TCGA) ccRCC database demonstrated that a low PFDN3 expression level correlates with poor survival in patients with missense-mutant VHL [92]. Thus, VHL mutations that disrupt prefoldin function lead to imbalances in VHL proteostasis, which promotes tumorigenesis.

Class 2 mutations of VHL have been shown to directly destabilize the protein *in vitro*, which promotes unfolding and aggregation [92, 94]. Some of these mutations, i.e., F136L and F119L, exist in the core of the protein, which disrupts a required aromatic tetrahedron structure required for stability [94]. In the cell, Hsp90 recognizes these misfolded intermediates and, with HOP and Hsp70, mediates their degradation [94, 97]. Class 3 mutations prevent the chaperonin TRiC from binding to VHL, leading to misfolded/unfolded VHL that is sent to the degradation pathway. A distinct, 55 amino-acid region that corresponds closely to exon 2 of VHL is both necessary and sufficient for binding to TRiC [96]. Loss of exon 2 leads to sporadic RCC, and approximately half of all VHL tumor mutations have been found to occur here [96, 102, 103]. Class 4 mutations of VHL prevent association with binding partners elongins B and C, leaving monomeric VHL unstable, aggregate-prone, and rapidly degraded [94, 95]. Pathogenic mutations, such as L158P, are commonly found in the region of VHL that binds to elongin C (amino acids 157–172), compromising VHL protein stability [89, 95, 96, 104]. Taken together, it is clear that the chaperone network is integral for the function of VHL.

### Chaperoning of the tumor suppressor tuberous sclerosis complex

Tuberous sclerosis complex (TSC) is an autosomal dominant disorder caused by inactivating mutations in either tumor suppressor *TSC1* or *TSC2* [105]. Patients develop hamartomas throughout the body, including the brain, skin, and kidneys, which may progress to malignancy or directly lead to severe neurologic complications such as epilepsy and autism [105]. The *TSC1* and *TSC2* genes encode the proteins Tsc1 (hamartin) and Tsc2 (tuberin), respectively, which form a functional complex in the cell (Tsc1/2) [105, 106]. The primary function of Tsc1/2 as a tumor suppressor is to inhibit cell growth and proliferation by antagonizing the mammalian target of rapamycin (mTOR) pathway, a signaling network that is a regulatory hub for cell growth [107, 108]. Loss of mTOR inhibition by Tsc1/2 results in unregulated cell growth which is reflected by reduced neuronal ciliation and the presence of giant cells within hamartomas from TSC patients [107, 109].

**Figure 3:**
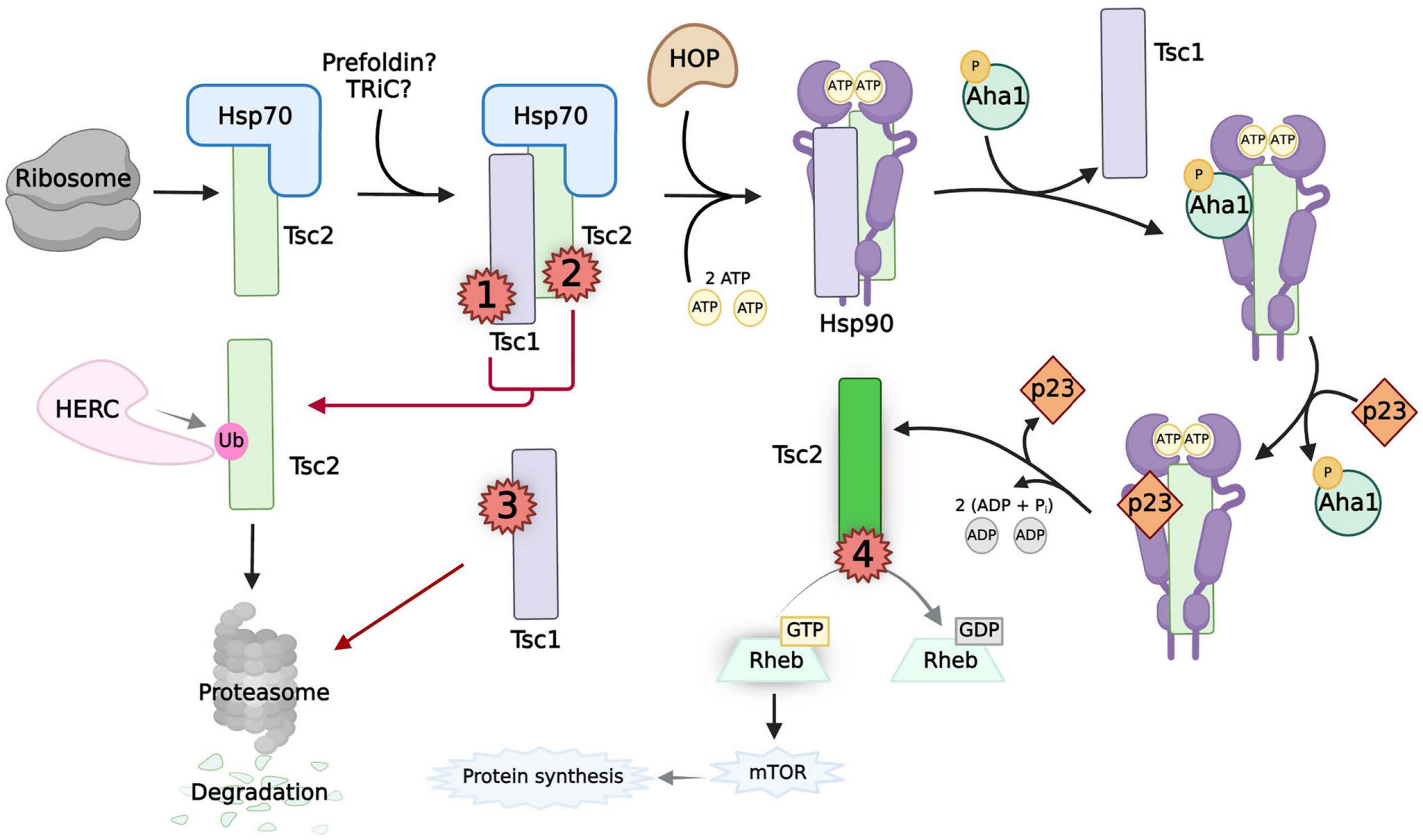
The chaperoning, activity, and pathogenic mutations of TSC. Black arrows indicate the normal chaperoning of Tsc2. Naϊve Tsc2 (light green box) is chaperoned by Hsp70, possibly with the help of prefoidin and TRiC, to Tsc1. The cochaperones of Hsp90, TSC1 and HOP, mediate transfer to Hsp90. Interactions between TSC1, phosphorylated Aha1, and p23 control the Hsp90 ATPase cycle to allow Tsc2 to fold into its native state (neon green box). Active Tsc2 acts as a GAP to regulate mTOR activity. Red arrows indicate pathways caused by multiple classes of pathogenic mutations on Tsc1/2 (red numbers). Class 1 mutations on Tsc1 and Class 2 mutations on Tsc2 disrupt the Tsc1:Tsc2 complex. This results in unbound, naϊve Tsc2 to become ubiquitinated by HERC for proteasomal degradation. Class 3 mutations on Tsc1 destabilize the protein before interaction with Tsc2, resulting in proteasomal degradation of both Tsc1/2. Class 4 mutations occur in the GAP domain of Tsc2, rendering it non-functional.

Mechanistically, mTORC1 supports biomass generation by enhancing protein translation efficiency as well as increasing the production of ribosomes and nucleotide precursors in response to growth factors, cellular energy and nutrient levels [108]. Tsc2 functions as a GTPase Activating Protein (GAP) toward Rheb, the small GTPase upstream of mTOR (Figure 3) [110]. Tsc2 inactivates Rheb by inhibiting conversion from the GDP-bound (inactive) to GTP-bound (active) form, thereby turning mTORC1 off [110–112]. Tsc1 is necessary for the function of Tsc2 by protecting it from degradation, as the overexpression of Tsc1 is able to raise the levels of Tsc2 in the cell, and the loss of Tsc1 significantly decreases the levels of Tsc2 [55, 113–115]. In line with this, the phosphorylation of downstream targets of mTOR—such as eukaryotic translation initiation factor 4E-binding protein 1 (4E-BP1) and ribosomal protein S6 kinase beta-1 (S6K1)—are inhibited when either Tsc1/2 are overexpressed [116]. It follows that functional Tsc1 is required in the cell for the inhibition of mTORC1, but, Tsc2 alone inhibits Rheb GTP levels [112, 116]. Therefore, abnormal mTOR activation via loss of functional Tsc1 or Tsc2 promotes oncogenesis by maintaining the necessary cellular signals for tumor growth, survival, and proliferation [108].

The stability and activity of Tsc2 is tightly regulated by a balance between molecular chaperones and the E3 ubiquitin ligase HERC1, which is responsible for ubiquitinating Tsc2 to promote its degradation [54]. Notably, Tsc1 and HERC1 compete for binding to the NH2 terminal domain of Tsc2 [54]. Tsc1 binds to Tsc2 more strongly than HERC1, shielding the tumor suppressor from proteasomal degradation by only allowing HERC1 to bind to free Tsc2 [54]. Hsp70 is also necessary to prevent the ubiquitination and proteasomal degradation of Tsc2, presumably by chaperoning newly-synthesized Tsc2 from the ribosome to Tsc1 (Figure 3) [55]. Previous work has demonstrated that the cochaperone complex R2TP/Prefoldin-like (R2TP/PFDL) complex interacts with both Tsc1/2; however, the exact contribution of this complex to the stability of Tsc2 is unknown [117]. Importantly, Tsc1 also acts as a cochaperone of Hsp90 to maintain the function of Tsc2, explaining its role in protecting Tsc2 from degradation [55]. Mechanistically, Tsc1 acts as a loading scaffold for Tsc2 to facilitate the direct binding of Tsc2 to Hsp90 [55]. Notably, the cochaperone Aha1 competes with Tsc1 for the same binding sites in the middle domain of Hsp90. Tsc1 has a stronger affinity for Hsp90, unless Aha1 is phosphorylated at the Y223 residue [50, 55]. Phospho-Y223-Aha1 displaces Tsc1 from Hsp90, leaving Tsc2 in the chaperone complex, and allows progression of the chaperone cycle [50, 55]. The cochaperone p23 was found to interact with Tsc2 at this stage of the Hsp90 cycle, where it stabilizes the closed conformation of Hsp90 [47, 55]. This complex likely remains until Tsc2 is matured and able to inhibit Rheb activity. This chaperoning by Hsp90 is essential to prevent proteasomal degradation of Tsc2 [55]. Therefore, the correct chaperoning of Tsc2 is crucial for its activity, and, consequently, the control of mTOR and prevention of oncogenesis.

### Pathogenic mutations in *TSC*

Like other tumor suppressors, there are multiple classes of pathogenic mutations found in both *TSC1* and *TSC2* that contribute to disease (Figure 3). Exons 34–38 encode the GAP-domain of Tsc2 at the C-terminus [118]. TSC patients often present with truncating mutations as well as, more infrequently, missense point mutations in the GAP domain such as V1571H, causing abrogation of the GAP activity of Tsc2 [110, 113, 118, 119]. These mutations, (Figure 3, Class 4), allow for the full maturation of the Tsc2 through the chaperoning network, but prevent correct GAP activity toward Rheb. However, the other classes of mutations in *TSC1/2* cause the TSC phenotype and/or metastasis by dysregulating the chaperoning of Tsc2 [54, 55, 120–123].

Pathogenic mutations that disrupt the formation of the Tsc1:Tsc2 complex (Classes 1–3) prevent proper chaperoning of Tsc2 [54, 55, 120–123]. Class 2 mutations within *TSC2* can be truncating or missense point mutations such as R611Q [54, 120–122]. These mutants have a weaker affinity for Tsc1, yet retain their ability to bind to HERC1, and as such are ubiquitinated and rapidly degraded through the proteasome [54, 120]. Subsequently, Tsc2 loss causes hyperactivation of Rheb and the phosphorylation of the downstream targets of mTOR [54, 120–122]. Mutations of *TSC1* fall into either Class 1 or Class 3 categories. Class 1 mutations include truncation or point mutations that disrupt Tsc1/2 binding. In *TSC1*, exons 17–18 encode the region responsible for binding to Tsc2, and multiple pathogenic missense mutations are found here that result in abrogated mTOR inhibition [118, 122]. Class 3 mutations found in *TSC1* result in the loss of Tsc1 expression by deletions or NH2-terminal nonsense, frame-shift, or missense mutations [123–126]. Some of these pathogenic Class 3 mutations on Tsc1, such as L117P, do not occur within the Tsc2-binding or Hsp90-binding domains [55]. However, these Tsc1 mutants are highly unstable, and their susceptibility to proteasomal degradation prevents Tsc2 binding to Hsp90, a critical step in the maturation of functional Tsc2 [55, 123, 126]. Thus, chaperones are central in the regulation of Tsc1/2 and the prevention of oncogenesis.

### The specialized cochaperones FNIP1/2 protect the tumor suppressor folliculin from degradation

**Figure 4:**
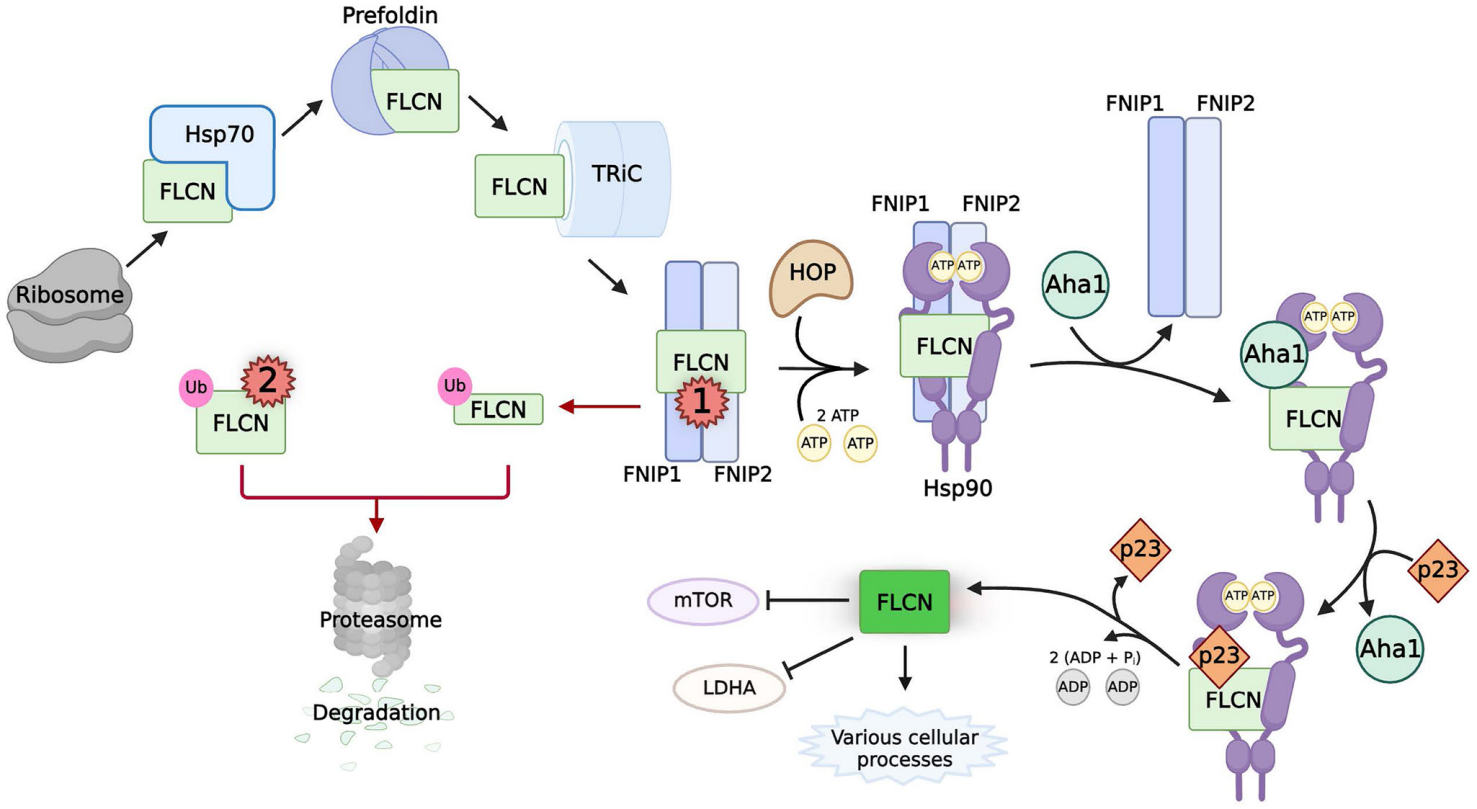
The chaperoning, activity, and pathogenic mutations of FLCN. Black arrows indicate the normal chaperoning of FLCN. Naϊve FLCN (light green box) is chaperoned by the sequential interactions of Hsp70, prefoldin, and TRiC before binding to FNIP1/2. The cochaperones of Hsp90, FNIP1/2 and HOP, mediate transfer to Hsp90. Interactions between FNIP1/2, phosphorylated Aha1, and p23 control the Hsp90 ATPase cycle to allow FLCN to fold into its native state (neon green box). Active FLCN is involved in many cellular processes, including inhibiting LDHA activity and mTOR activity. Red arrows indicate pathways caused by multiple classes of pathogenic mutations on FLCN (red numbers). Class 1 mutations of FLCN prematurely truncate the protein, disrupting the interaction between FNIP1/2. Unbound, truncated FLCN is ubiquitinated for proteasomal degradation. Class 2 mutations on FLCN are missense and deletion mutations that result in the ubiquitination and degradation of the protein through unknown mechanisms.

Germline mutations in the tumor suppressor *FLCN* cause Birt-Hogg-Dubé (BHD) syndrome, which is characterized by benign skin lesions, pulmonary cysts, spontaneous pneumothorax, as well as chromophobe, clear cell, oncocytoma, and hybrid oncocytic RCC [61, 127, 128]. The proposed functions of the FLCN protein are varied, and its activity has been implicated in a diverse number of processes throughout the cell. These include regulation of anaerobic glycolysis (as an endogenous inhibitor of lactate dehydrogenase A), nutrient sensing and autophagy (through GAP activity toward Rag C/D and as a negative regulator of AMPK and mTOR), control of mitochondrial biogenesis (by suppressing PGC1α), and ribosomal RNA biogenesis (by disrupting Rpt4 binding to the rDNAlocus) [129–136]. FLCN forms a complex with folliculin-interacting proteins 1 and 2 (FNIP1 and FNIP2) (Figure 4), and kidney-specific double homozygous inactivation of *FNIP1/2* or *FLCN* in mice results in enlarged multi-cystic kidneys [135, 137–139].

The stability of FLCN is maintained in the cell by chaperones (Figure 4). Inhibition of Hsp70 or Hsp90 results in the ubiquitination and proteasomal degradation of FLCN [56]. FLCN also interacts with many established Hsp70 and Hsp90 cochaperones, such as HOP, p23, and Aha1 as well as members of the TRiC complex [56]. Thus, newly synthesized FLCN is chaperoned from the ribosome to its binding partner FNIP1/2 by Hsp70 and TRiC. The stability of FLCN depends on this binding to FNIP1/2, which exist as a homodimer and heterodimer, as the silencing of both FNIPs significantly decreases the stability of FLCN [56, 138, 139]. The FNIPs act as cochaperones of Hsp90, with their function required for the ‘loading’ of FLCN to the chaperone [56]. HOP also likely mediates the formation of the FLCN:FNIP:Hsp90 complex. Interestingly, the FNIPs were also found to inhibit the ATPase activity of Hsp90 [56]. This is in opposition to Aha1, which stimulates Hsp90 ATPase activity. Aha1 and the FNIPs compete for binding to Hsp90 to fine tune the speed of its chaperone cycle, with Aha1 able to displace either FNIP from Hsp90 [56]. The cochaperone p23 likely plays a role here, since it stabilizes the closed conformation of Hsp90 [47, 56]. This would allow time for the maturation of FLCN in complex with Hsp90 before its release to perform its various functions throughout the cell [129–136]. Of note, an additional cellular function of FNIP1 has been recently identified as a negative regulator of angiogenesis [140]. This warrants further investigation, given that this complex chaperone network is necessary to maintain functional levels of mature FLCN protein to prevent oncogenesis.

### *FLCN* pathogenic mutations and proteasomal degradation

Approximately 93% of all pathogenic *FLCN* mutations result in a prematurely truncated protein (Figure 4, Class 1) [141]. These mutations abrogate the FNIP binding domain of FLCN protein [137, 142] and therefore disrupt the association of FLCN with Hsp90 leading to its instability [56]. Without chaperoning by Hsp90, elevated FLCN turnover results in pathogenesis. Many of the rarer missense or single nucleotide deletion pathogenic mutations of FLCN cause severe misfolding, leaving them aggregation-prone and subject to degradation by the proteasome (Figure 4, Class 2) [143, 144]. However, some missense pathogenic mutations of FLCN remain stable, falling outside of Class 1 or Class 2 mutations [144]. Such mutations, like FLCN-K508R, could potentially directly impact FLCN function rather than stability, but further characterization is required [144]. Outstanding questions also include those about the mechanism of FLCN ubiquitination. Which E3 ubiquitin ligase is involved? What cellular signals regulate this process? Given the multiple emerging functions of FLCN, this is an important subject of active research.

## COCHAPERONE COMPENSATION AND CROSSTALK

Recent evidence has uncovered that extensive overlapping of the cochaperone/chaperone network allows for functional compensation that has a clinically meaningful impact. One such example was found in an unusual renal angiolipoma (AML)—typically associated with TSC syndrome—in a Birt-Hogg-Dubé (BHD) patient harboring a truncating *FLCN* mutation [145]. The instability of this reported FLCN mutation was caused by the loss of interaction with FNIP1 (described in Figure 4). However, this truncated FLCN retained its ability to bind to Hsp90 and bound more to Tsc1. Overexpression of Tsc1 stabilized the expression of the mutant, suggesting that this cochaperone, traditionally thought to only stabilize Tsc2, can compensate for the loss of another [145]. Notably, the Tsc1-mediated stabilization of the mutant FLCN to compensate for FNIP1 resulted in the destabilization of Tsc2 [145]. Subsequent dysregulation of the mTOR pathway caused the development of the rare AML in this BHD patient [145]. Evidence for this mechanistic crosstalk has also been demonstrated in mice, where silencing of *FNIP1* synergized with silencing of *TSC1* to activate mTOR and accelerate renal cyst formation [146]. Additionally, the absence of Tsc1 in mouse embryonic fibroblast cells was found to significantly reduce the stability of FLCN [55].

Taken together, these data indicate that even though compensation may occur, all components of the chaperone system need to be functional to maintain proteostasis.

Interestingly, mutations in *TSC1* (such as Classes 1 and 3, Figure 3) are associated with a less severe disease phenotype [118, 147]. This is consistent with the function of Tsc1 as a cochaperone of Hsp90, as multiple mTOR pathway components are clients of Hsp90 [12]. The loss of Tsc1 causes destabilization of Tsc2, which may allow elevated mTOR activity, but the other Hsp90 clients in the mTOR pathway would also be destabilized. This could attenuate the effect of Tsc1 loss compared to Tsc2 [12, 148]. This example of complicated crosstalk between cochaperones of tumor suppressors is likely not unique, as multiple tumor suppressors have been identified as Hsp90 associated cochaperones or clients, such as p53, BDC2, and LKB1 [12, 149–152]. Notably, the Tsc1, FNIP1 and FNIP2 cochaperones were found to form heterocomplexes in cells which fine-tuned Hsp90 client activity [153]. This underscores the central role that chaperones play in the regulation of tumor suppressors and the prevention of oncogenesis, warranting further research to dissect exact molecular mechanisms involved in pathogenesis.

## CONCLUSIONS AND FUTURE PERSPECTIVES

The chaperone network is a complicated system that includes many components, such as chaperones, cochaperones, chaperonins and clients themselves. Disruption of tumor suppressor chaperoning is the mechanism of pathogenicity of many patient-associated mutations in several genes involved in RCC [54–56, 94–97, 120–123, 137, 142]. Mounting evidence suggests that this is not a unique feature of RCC, but that multiple classes of these chaperone-disrupting pathogenic mutations exist in other cancers as well [154–158]. Additional research on these regulatory pathways could provide insight into currently uncharacterized mechanisms of tumorigenesis.

Additionally, the complexity of the chaperone network is furthered by post-translational modifications (PTMs). These have important roles in the regulation of the chaperone pathway. For example, PTMs of Hsp90 determines its binding to cochaperones, and, conversely, PTMs on cochaperones—like Aha1—determines its binding to Hsp90 [21, 50]. Many PTMs on tumor suppressors impact their stability and function [159]. However, a detailed understanding of how these pathways are regulated is lacking. Determining the factors that influence tumor suppressor regulation will clarify how normal cells can enter the early stages of malignant transformation.

Although tumor suppressor function is protected by molecular chaperones, many oncoproteins are also clients of the chaperone network [160]. Accordingly, the overexpression of Hsp90, Hsp70, and Hsp60 have been shown to promote tumor growth and metastasis in multiple cancers [160–163]. Because of this, a large body of research has been dedicated to developing chaperone inhibitors as anticancer therapeutics (reviewed elsewhere [161, 164, 165]). Although in the RCCs discussed above the inability of chaperones to stabilize mutated tumor suppressors is driving oncogenesis [54, 55, 94, 99, 120–123, 137, 142–144, 154–158], a growing body of evidence suggests that continued oncogenic proliferation is heavily reliant on chaperone function, and that chaperone inhibition specifically causes cancer cell death [160, 166–169]. Hsp90 and Hsp70 inhibitors promote apoptosis through a variety of mechanisms including downregulating the pro-survival Akt kinase, promoting the migration of pro-apoptotic proteins Bax and Bad, and allowing the formation of the death inducing signaling complex [160, 166–169]. Importantly, Hsp90 inhibitors preferentially accumulate in tumor cells, mitigating unwanted side effects in normal cells [170–172]. Due to the similar expression profiles and functions of Hsp90 and Hsp70 in cancer, it is possible that Hsp70 inhibitors also accumulate selectively in tumor cells through a similar mechanism (although further research on this topic is necessary) [160, 162, 163]. Given the tumor-specific ability of Hsp90 inhibitors to induce apoptosis, Hsp inhibition could be a therapeutic approach for the RCCs discussed above—regardless of the initiating oncogenic event being dysregulated tumor suppressor chaperoning. Alternatively, modulating chaperone activity instead of fully abolishing it also has therapeutic potential in these cancers. One can imagine “fine-tuning” chaperones to protect tumor suppressor proteins while allowing the degradation of oncoproteins. However, more extensive knowledge on the impact of co-chaperones and PTMs on chaperone activity is necessary to achieve this goal. Computational approaches, in conjunction with detailed structural information gained from classic biochemical experiments, have been increasingly useful in understanding the complexity of how these factors may affect one another [173–177]. Such future research, perhaps also with the aid of artificial intelligence, will be instrumental in the effort to appropriate the chaperone network for cancer therapy.

Overall, it is clear that oncogenesis can result from the dysregulation of tumor suppressor stabilization by chaperones. This mechanism of pathogenesis is distinct from the large body of research that focuses solely on cellular pathways specific to each tumor suppressor gene. Focusing on the holistic lifecycle of these proteins reveals commonalties between the widely diverse group of tumor suppressors, which is invaluable to inform therapy development for multiple cancers.
